# Down-regulated GATA-1 up-regulates interferon regulatory factor 3 in lung adenocarcinoma

**DOI:** 10.1038/s41598-017-02700-5

**Published:** 2017-05-31

**Authors:** Lu-Lu Wang, Zheng-Sen Chen, Wen-Di Zhou, Jin Shu, Xiao-Hua Wang, Rui Jin, Li-Li Zhuang, Mir Alireza Hoda, Hao Zhang, Guo-Ping Zhou

**Affiliations:** 1 Dpartment of Pediatrics, The First Affiliated Hospital, Nanjing Medical University, Nanjing, Jiangsu Province China; 2 Department of Urology, The Second Affiliated Hospital, Nanjing Medical University, Nanjing, Jiangsu Province China; 3 Dpartment of Pediatrics, Huai’an First People’s Hospital, Nanjing Medical University, Huai’an, Jiangsu Province China; 4 Department of Pediatric Respiration, Affiliated Wuxi People’s Hospital, Nanjing Medical University, Wuxi, Jiangsu Province China; 5 Dpartment of Pediatrics, Nanjing First Hospital, Nanjing Medical University, Nanjing, Jiangsu Province China; 6 Translational Thoracic Oncology Laboratory, Division of Thoracic Surgery, Department of Surgery, Comprehensive Cancer Center, Medical University Vienna, Vienna, Austria; 7 Department of Thoracic and Cardiovascular Surgery, Affiliated Hospital of Xuzhou Medical University, Xuzhou, Jiangsu Province China

## Abstract

Interferon regulatory factor 3 (IRF-3) is widely known for its prompt response against viral infection by activating the interferon system. We previously reported that E2F1, Sp1 and Sp3 regulated transcriptional activity of IRF-3. Recently, different expression patterns of IRF-3 were found in lung cancer, leading to the alternation of the immunomodulatory function in tumorigenesis. However, the mechanism of transcriptional regulation of IRF-3 in lung cancer has not been extensively studied. Here, we investigated the characterization of IRF-3 promoter and found that GATA-1 bound to a specific domain of IRF-3 promoter *in vitro* and *in vivo*. We found elevated IRF-3 and decreased GATA-1 gene expression in lung adenocarcinoma in Oncomine database. Additionally, higher IRF-3 gene expression was observed in human lung adenocarcinoma, accompanied by aberrant GATA-1 protein expression. We further analyzed the relationship of GATA-1 and IRF-3 expression in lung adenocarcinoma cell lines and found that inhibition of GATA-1 by siRNA increased the promoter activity, mRNA and protein levels of IRF-3, while over-expression of GATA-1 down-regulated IRF-3 gene expression. Taken together, we conclude that reduced GATA-1 could be responsible for the upregulation of IRF-3 in lung adenocarcinoma cells through binding with a specific domain of IRF-3 promoter.

## Introduction

Lung cancer is the leading cause of death related to cancer, and is diagnosed in more than 1.6 million new patients annually^1^. The majority of patients suffering from lung cancer are diagnosed with non-small-cell lung cancer (NSCLC), among which lung adenocarcinoma represents the predominant subtype. There is accumulating evidence that the development of lung adenocarcinoma is associated with several genetic and epigenetic alterations in oncogenes and suppressor genes^2^.

IRF-3 is a transcription factor that plays important regulatory roles in the interferon (IFN) system in response to virus infection. IRF-3 exists in the cytoplasm as a latent form in resting cells. Upon virus infection, IRF-3 is activated by the phosphorylation of Ser/Thr residues in its C-terminus and is translocated to the nucleus to initiate the transcription of numerous innate immune genes, including type I IFN. Then, type I IFN acts in an autocrine and paracrine manner to amplify the cascades of IFN stimulated gene synthesis. Previous studies demonstrated that type I IFN contributed to anti-tumor therapy^3^. Recently, much progress has been made in understanding the strong regulation of IRF family in tumor pathogenesis, DNA damage response and cell cycle^4,5^. Loss-of-function mutations of IRF-1, IRF-4, IRF-5, IRF-7 and IRF-8 gene expression have been previously reported in several human cancers, including lung, hematopoietic, hepatocellular, gastric, pancreatic, myeloid and lymphoid cancers^6–10^. It was recently reported that IRF-3 might function as a tumor suppressor in tumorigenesis by inhibiting growth of cancer cell *in vitro* and *in vivo*. Kim *et al*. reported that ectopic over-expression of IRF-3 induced p53-dependent cell proliferation inhibition and cellular senescence in both normal and cancer cells^11–13^. Duguay *et al*. demonstrated that IRF-3 could enhance cytokine production and recruit inflammatory cells to the site of B16 melanoma^14^. Thus, elucidating the molecular mechanism underlying the transcriptional activation of IRF-3 gene may yield insights into how IRF-3 was involved in lung adenocarcinoma.

In this study, we found a specific GATA-1 binding site in IRF-3 promoter and observed aberrant gene expression of IRF-3 and GATA-1 in lung adenocarcinoma. We further investigated their relationship in lung adenocarcinoma cell lines and found that suppression of endogeneous GATA-1 increased the IRF-3 transcriptional activity through the GATA-1 element in the IRF-3 promoter.

## Results

### Deletion of GATA-1 binding site leads to an increase of IRF-3 promoter activity

**Figure 1:**
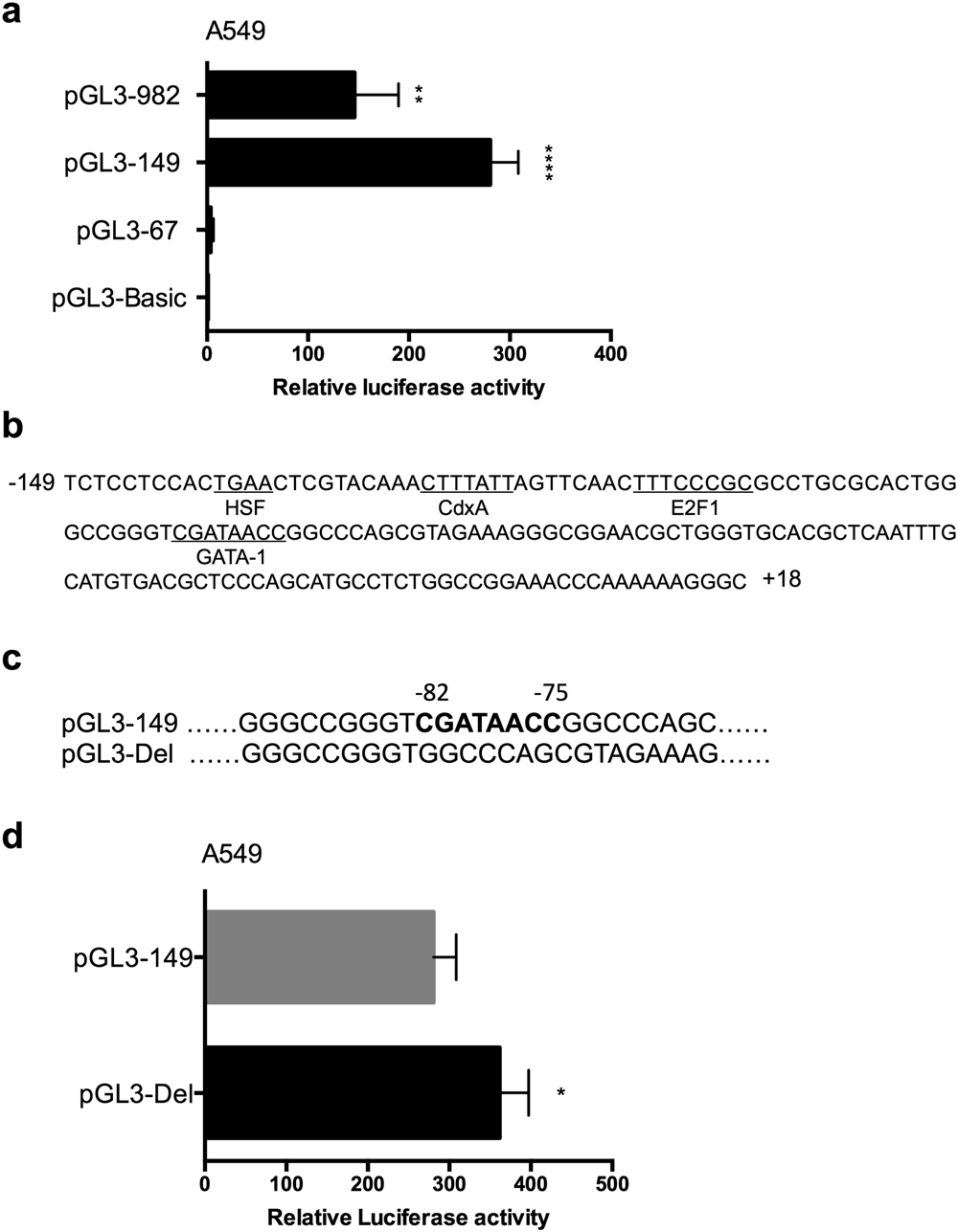
Functional analysis of putative GATA-1 element in IRF-3 promoter. (**a**) Relative luciferase activities of truncated plasmids of IRF-3 promoter. (**b**) Putative consensus binding sites of transcription factors were identified by TFSEARCH ver.1.3 and Matlnspector. (**c**) Nucleotide sequences of the original and GATA-1 element deleted version of pGL3-149 plasmid (pGL3-Del). (**d**) The promoter activity of pGL3-149 and pGL3-Del in A549 cells. Error bars represent the mean ± S.D. (n = 3). Statistical analyses were done using Student’s-t tests. *p < 0.05; **p < 0.01; ****p < 0.0001 compared with control.

It is reported that E2F1, Sp1 and Sp3 transcription factors directly binds to IRF-3 gene promoter and regulate its transcriptional activity^15,16^. To further identify the promoter activity of IRF-3 in A549 cells, we cloned truncated plasmids of human IRF-3 promoter as performed as before^15,16^ and transfected pGL3-982, pGL3-149, pGL3-67 and pGL3-Basic vectors into A549 cells. Luciferase assays revealed that pGL3-982 and pGL3-149 had higher promoter activity in A549 cells. Deletion of the DNA fragment from −149 to −67 caused a significant reduction in luciferase activity of IRF-3 promoter (Fig. 1a). This suggested a potential major regulatory element in this region. To further identify transcription factors involved in this region, we used the TFSEARCH ver. 1.3 and Matlnspector to search for the functional minimal promoter pGL3-149 for potential target sites and found that this region contains a consensus GATA-1 element (−82 to −75), HSF element (−139 to −136), CdxA element (−125 to −119) and E2F1 element (−110 to −103) (Fig. 1b). According to the result shown above, the plasmid pGL3-149 was the minimal functional promoter containing a GATA-1 element. To determine the function of GATA-1 binding site in IRF-3 promoter, we constructed a GATA-1 element deletion version of pGL3-149, named pGL3-Del (Fig. 1c). As shown in Fig. 1d, disruption of GATA-1 element led to an amelioration of IRF-3 promoter activity. Thus, our results indicated that GATA-1 element was important in maintaining the promoter activity of minimal IRF-3 promoter.

### GATA-1 binds *in vitro* and *in vivo* to IRF-3 promoter

**Figure 2:**
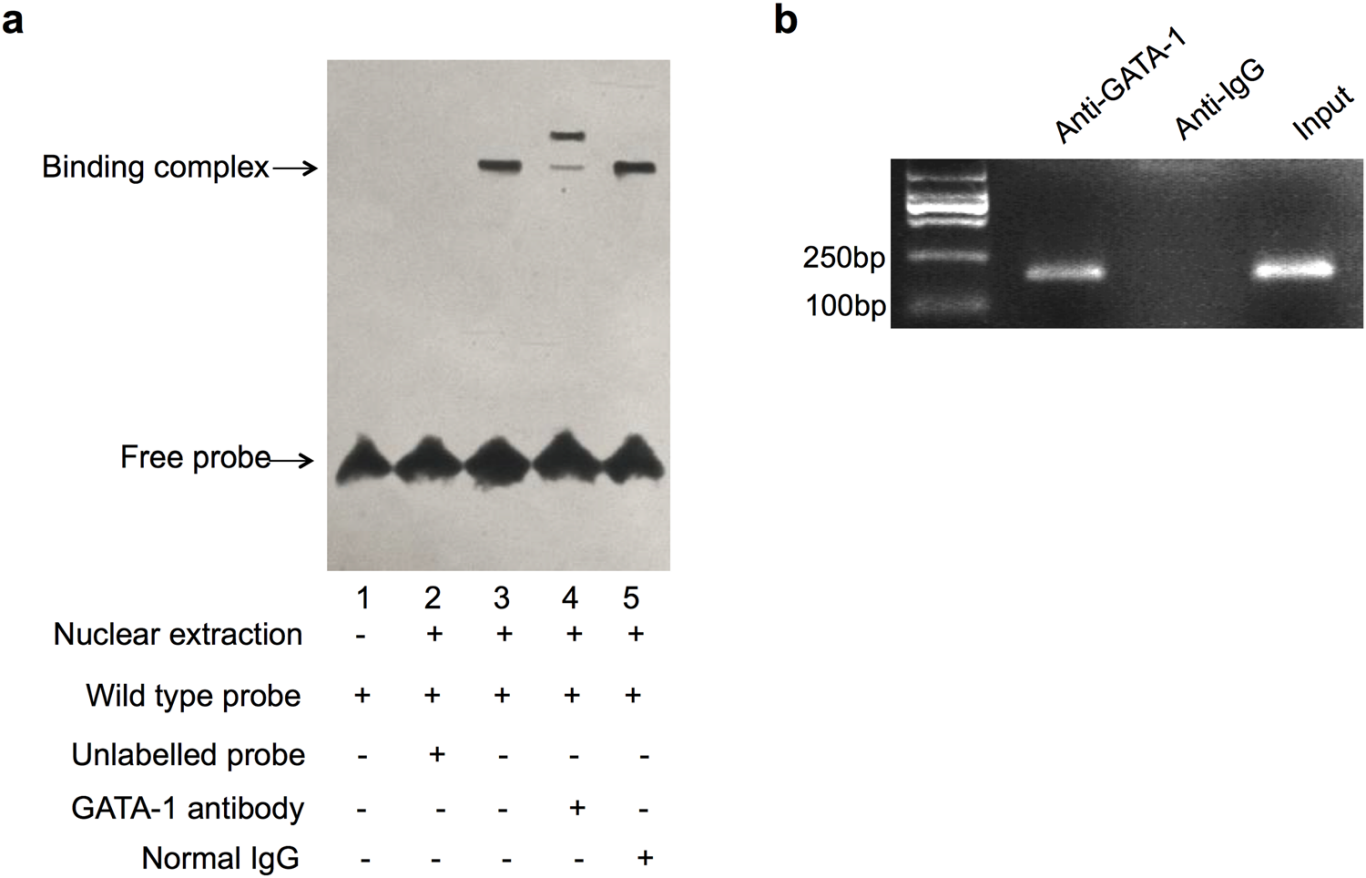
The interaction of GATA-1 and IRF-3 promoter *in vitro* and *in vivo*. (**a**) EMSA was performed with a DNA probe harboring the GATA-1 element (−82 to −75) on the IRF-3 promoter as described in the methods section. (**b**) A549 cell lysate was subjected to ChIP assay using anti-GATA-1 (IP: GATA-1) or non-specific IgG (negative control) antibodies. After purification of DNA, the IRF-3 promoter area (−149 to +18) was amplified by semi-quantitative PCR. The full-length gel was presented in Supplementary Figure S1.

To investigate whether GATA-1 binds to the putative binding site (−82 to −75) *in vitro*, we performed electrophoretic mobility shift analysis (EMSA) with A549 cells nuclear extracts. We constructed a wild type probe by combining nuclear protein with labeled wild type oligonucleotides (lane 2, Fig. 2a). Competing with unlabeled probes significantly reduced the formation binding complex (lane 3, Fig. 2a). A further supershift study showed that the binding complex was blocked when incubated with anti-GATA-1 antibody (lane 4, Fig. 2a).

To further confirm that GATA-1 binds to IRF-3 promoter *in vivo*, chromatin immunoprecipitation (ChIP) assay was performed. The cross-linked chromatin was sonicated and immunoprecipitated with anti-IgG and anti-GATA-1 antibodies, followed by PCR with primers flanking the GATA-1 binding site of IRF-3 promoter. As shown in Fig. 2b, binding of GATA-1 was detected at −149 to +18 region, where binding of non-specific IgG failed to be found. These data suggested that GATA-1 bound to IRF-3 promoter *both in vitro* and *in vivo*.

### Expression of IRF-3 and GATA-1 in human lung adenocarcinoma

**Figure 3:**
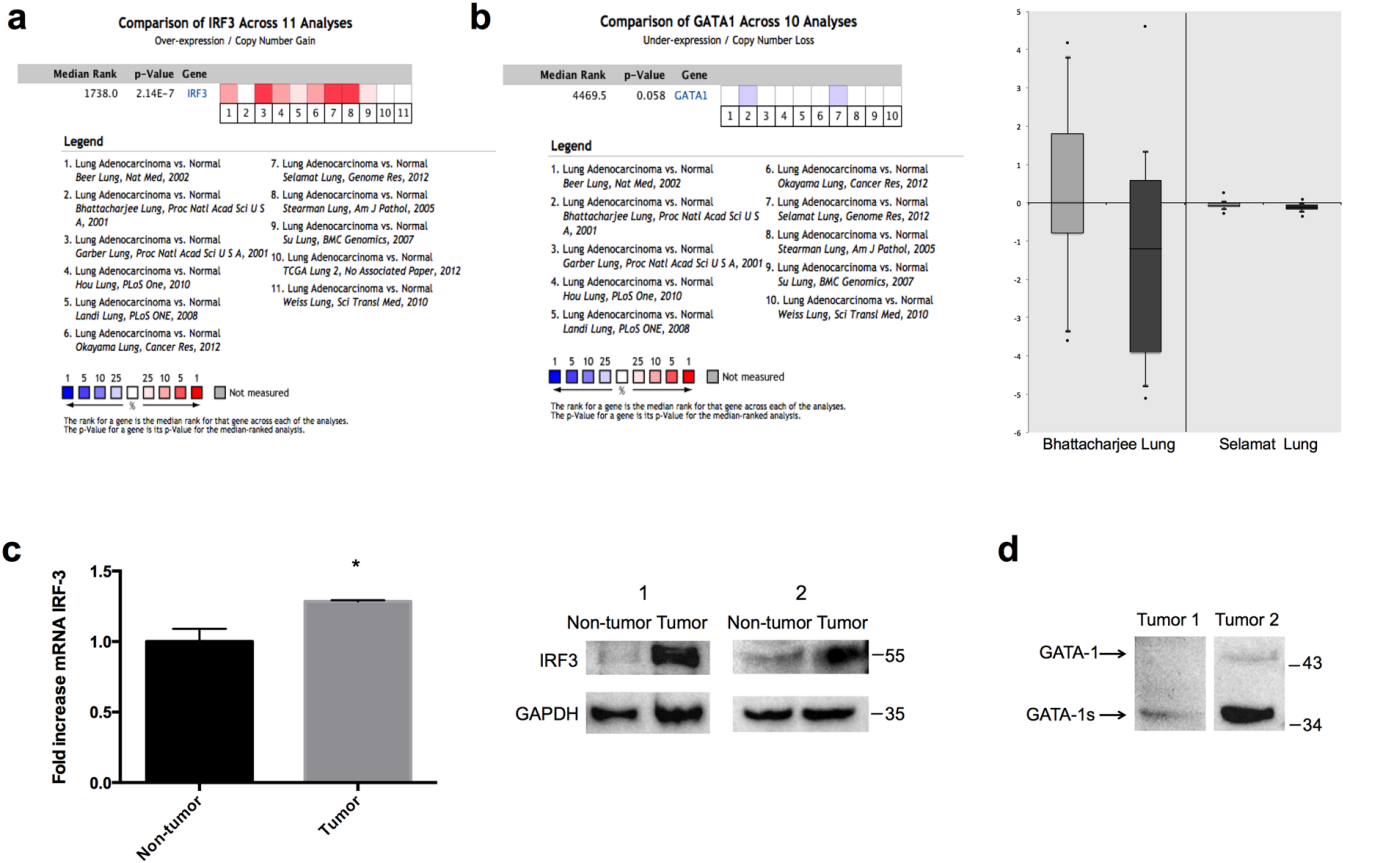
Upregulation of IRF-3 protein expression and down-regulated expression of GATA-1 protein in human lung adenocarcinoma tissue. (**a**) Eleven analyses were evaluated in comparing the RNA expression of IRF-3 between lung adenocarcinoma and normal tissue. Values above the average were considered IRF-3 over-expression (red). (**b**) Ten analyses of GATA1 gene demonstrated the different expression in lung adenocarcinoma compared to normal tissue. Lower expression of GATA1 was shown in two studies (blue). These two studies, Bhattacharjee Lung and Selamat Lung datasets studies, showed GATA-1 expression in normal tissues (light grey) and lung adenocarcinoma (dark grey). Log2 Median-Centered ratio was used to average all normal samples in data analysis. (**c**) The endogenous IRF-3 mRNA and protein expression was examined in lung adenocarcinoma and adjacent normal tissue. *P* values were determined by Student’s-t test (*p < 0.05). (**d**) The GATA-1 protein expression was detected in lung adenocarcinoma tissue by Western blot. GAPDH was used as a internal control. The full-length blots were presented in Supplementary Figure S2.

We conducted cDNA microarray analysis by using the Oncomine database to explore gene expression of IRF-3 and GATA-1 in lung adenocarcinoma. The Oncomine database was queried for IRF-3 and GATA-1 expression in lung adenocarcinoma and normal tissue. We used the search term “IRF-3” and isolated datasets representing lung adenocarcinoma and Cancer vs. Normal Analysis. We identified eleven datasets which contained IRF-3 DNA or RNA expression data. In the comparison analysis, eight datasets showed that IRF-3 RNA expression was over-expressed in lung adenocarcinoma compared to normal tissue (Fig. 3a). We then changed the search term into “GATA-1” and compared datasets representing lung adenocarcinoma and Cancer vs. Normal Analysis. Lower expression of GATA-1 RNA was found in lung adenocarcinoma compared with normal tissues in Bhattacharjee Lung and Selamat Lung datasets (Fig. 3b). These observations suggested that IRF-3 was up-regulated while GATA-1 was down-regulated in lung adenocarcinoma compared with normal tissues.

We further examined the expression of IRF-3 in human lung adenocarcinoma and adjacent normal tissue. As shown in Fig. 3c, IRF-3 mRNA and protein expression were significantly elevated in lung adenocarcinoma compared to adjacent normal tissue. We also observed aberrant expression of GATA-1 in lung adenocarcinoma. In one tissue from a patient with lung adenocarcinoma, a western blot experiment showed a lack of full-length GATA-1 protein expression and a weak signal for GATA-1 mutation (GATA-1s). In a tissue of another patient with lung adenocarcinoma, GATA-1s was highly expressed but the full-length GATA-1 was nearly absent (Fig. 3d). Considering GATA-1s was shown previously to be less functional than the full-length GATA-1^17^, these results demonstrated that suppression of endogenous GATA-1 gene expression might be associated with the up-regulation of IRF-3 in lung adenocarcinoma.

### Suppression of endogeneous GATA-1 enhances the IRF-3 transcriptional activity

**Figure 4:**
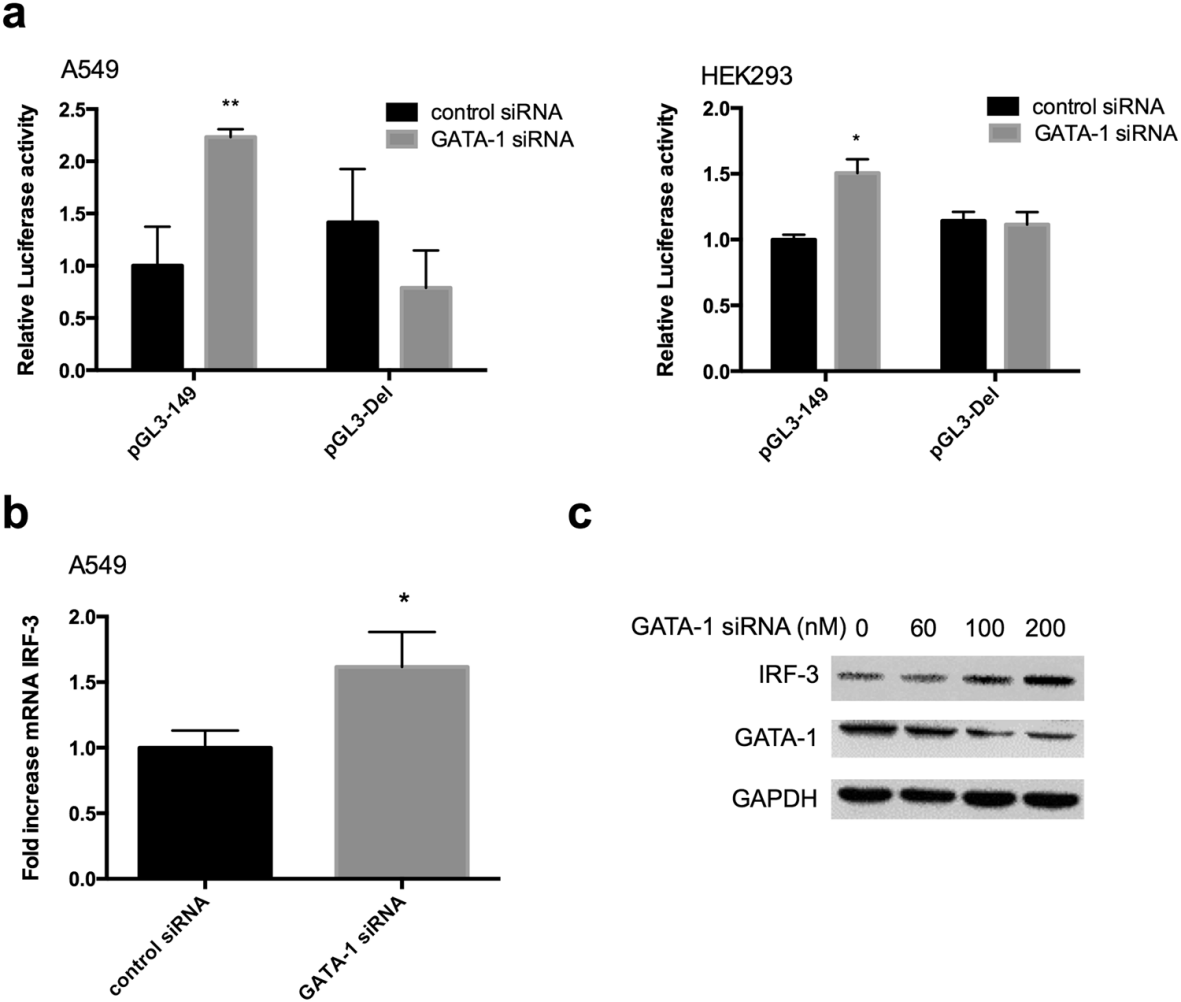
Inhibition of GATA-1 protein expression upregulates IRF-3 transcriptional activity. (**a**) pGL3-149 and pGL3-Del were transfected with GATA-1 siRNA or control siRNA into cells. Relative luciferase activity and (**b**) mRNA expression were detected at 24 h post-transfection. Statistical analyses were done using Student’s-t tests (*p < 0.05; **p < 0.01). (**c**) A549 cells were transfected with different dose of GATA-1 siRNA for 48 h. IRF-3 and GATA-1 proteins were analyzed by Western blot. The full-length blots were presented in Supplementary Figure S3.

**Figure 5:**
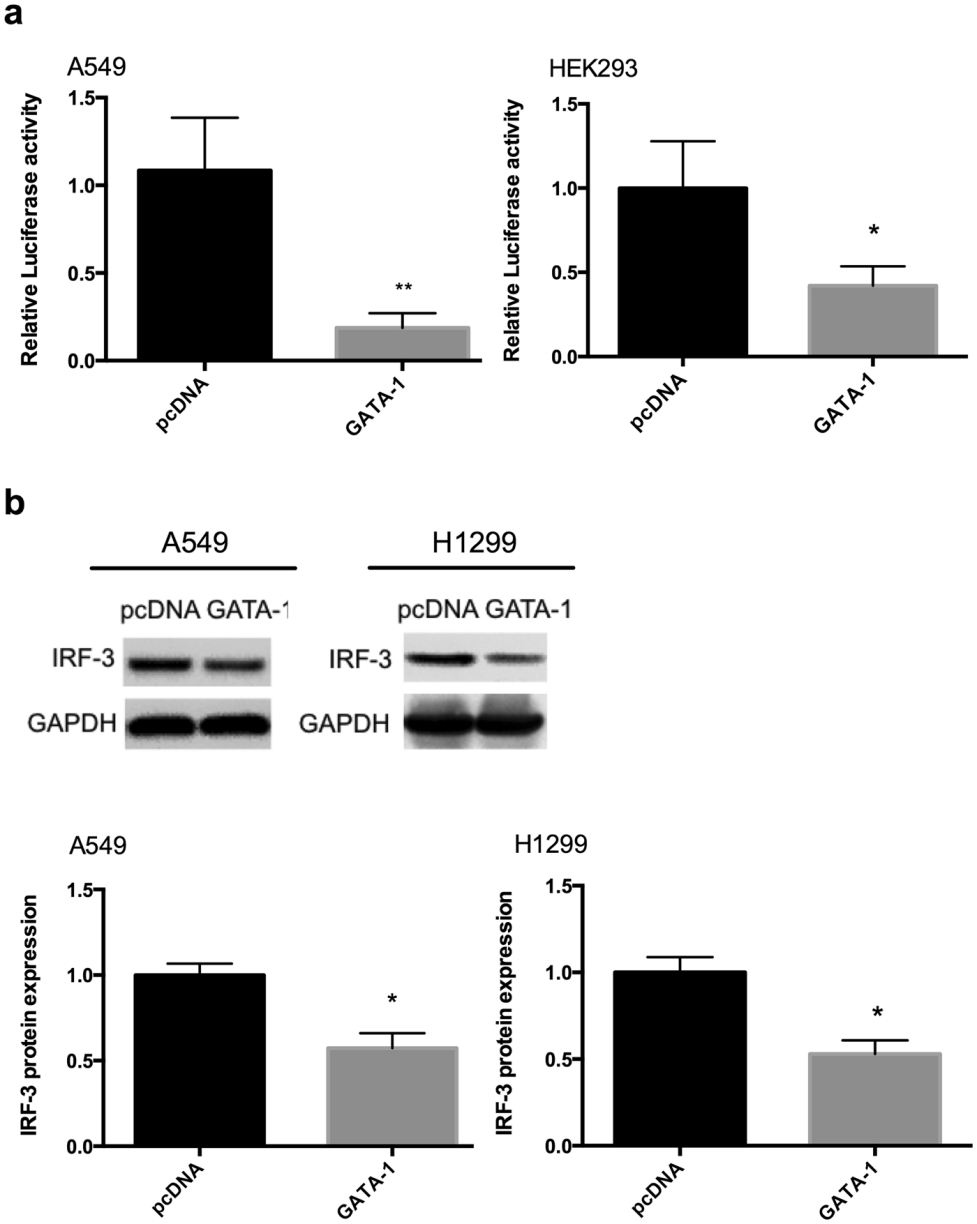
Over-expression of GATA-1 protein level decreases IRF-3 promoter activity. (**a**) pcDNA or pcDNA-GATA-1 plasmid was co-transfected with pGL3-149 into cells. (**b**) IRF-3 protein level was declined by overexpressed GATA-1 in different lung adenocarcinoma cell lines. GAPDH was used as a loading control. Error bars represent the mean ± S.D. from three independent experiments (*p < 0.05; **p < 0.01). The full-length blots were presented in Supplementary Figure S4.

To further address whether endogeneous GATA-1 deficiency directly influences IRF-3 gene expression, we performed small interfering RNA (siRNA) experiments in A549 cells. As shown in Fig. 4a, repression of endogenous GATA-1 induced an increase in promoter activity of IRF-3, but GATA-1 siRNA had no effect on the promoter activity without GATA-1 element. The experiment of GATA-1 siRNA showed that knockdown of GATA-1 increased IRF-3 mRNA expression (Fig. 4b) and led to a dose-dependent increase of IRF-3 protein (Fig. 4c). For the over-expression experiments, pcDNA or pcDNA-GATA-1 plasmid were co-transfected with pGL3-149 into cells. In contrast to the effect of suppression, GATA-1 over-expression caused a dramatic decrease of the promoter activity (Fig. 5a) and protein expression of IRF-3 in different lung adenocarcinoma cell lines (Fig. 5b).

## Discussion

GATA-1 contains two highly conserved zinc fingers, one of them being located at its C-terminal. This domain mediates GATA-1 binding to typical WGATAR elements of gene promoter^18^. We previously showed that a genomic fragment containing −149 to −93 of 5′ sequence flanking of IRF-3 promoter was the core transactivation region of IRF-3 promoter. GATA-1 element is necessary but not sufficient for maintaining the activation of IRF-3 promoter because deletion of −93 to −67 did not retain transcriptional activity^15,16^. However, in this study, we carried out the deletion of putative GATA-1 element −82 to −75 in wild pGL3-149 plasmid and identified that the deletion of this region restored the promoter activity (Fig. 1d). The binding ability of GATA-1 was also confirmed by *in vitro* with EMSA and *in vivo* with ChIP (Fig. 2). This observation is consistent with a previous report demonstrating that truncated plasmids of MTG16 promoter containing GATA-1 element did not retain the luciferase signal, while the GATA-1 element is still critical in regulating the transcription activation of the MTG16 gene^19^.

IRF-3 is essential for the anti-tumor pathogenesis in some cancer cells. Over-expression of IRF-3 inhibited cancer cell proliferation in a p53-dependent manner and ectopic expression of IRF-3 decreased B16 melanoma tumor growth *in vivo*^13,14^. But, little is known about IRF-3 transcriptional activity in lung adenocarcinoma. In our study, the expression levels of IRF-3 mRNA and protein were higher in lung adenocarcinoma tissue than in adjacent normal tissue (Fig. 3c), an observation which is consistent with our Oncomine analysis (Fig. 3a). Guo *et al*. have shown that IRF-3 was up-regulated in human NSCLC tissue^20^. A higher expression level of IRF-3 was also observed in the tumors of surviving patients with stage I NSCLC than that in patients who died from cancer^21^. Nevertheless, a study reported different expression patterns of IRF-3 in NSCLC patients, including both activation and suppression^22^. The inconsistency may derive from the diverse post-translational modification of IRF-3, e.g., phosphorylation and sumoylation, and different regulation of IRF-3 by cytokines and chemokines involved in the tumor microenvironment.

In Oncomine analysis, Bhattacharjee Lung and Selamat Lung datasets showed that GATA-1 gene expression was decreased in lung adenocarcinoma in comparison with normal lung tissues (Fig. 3b). In our experiments with human lung adenocarcinoma samples, we observed a higher protein level of GATA-1s and lack of full-length GATA-1 protein in lung adenocarcinoma tissue (Fig. 3d). The deficiency of GATA-1 in the tumor was accompanied by high expression of IRF-3, suggesting the potential relationship between decreased GATA-1 and increased IRF-3 in lung cancer cells. Thus, we addressed this question and found that suppressed endogenous GATA-1 protein expression increased the promoter activity, mRNA and protein of IRF-3 gene in lung adenocarcinoma cell lines. We also observed the similar regulation in HEK293 and HeLa cells (Supplementary Figure S5). To our knowledge, this is the first study demonstrating the low expression of GATA-1 and its significant role in regulating IRF-3 gene expression in lung adenocarcinoma cell lines. In humans, GATA-1 mRNA is alternatively spliced to deliver two protein isoforms, a full-length form GATA-1, and a short form from the mutations occurring in its second exon GATA-1s^23^. GATA-1s lacks the N-terminal domain leading to defects in cell proliferation and cell cycle, which is associated with hematopoietic disorders and Down syndrome in children. Accumulating studies have uncovered that GATA-1s is less active in trans-activating the downstream gene than the full-length GATA-1 in hematopoietic cell differentiation^17,24,25^. Our finding is beneficial for the exploration of mutated GATA-1 in non-hematopoietic disease.

Lung adenocarcinoma is a heterogeneous disease with dramatic genetic diversities in different patients. Due to few samples in this study, it is arbitrary to take a conclusion that GATA-1 was under-expressed and replaced by GATA-1s in lung adenocarcinoma. Gene sequencing is required to confirm the alternative splicing of GATA-1 in patients with lung cancer. Expression analysis in more patients is also required to elucidate the function of GATA-1 and mutated GATA-1 in lung adenocarcinoma. On the other hand, GATA-1s itself could play as a suppressor in the down-regulation of IRF-3 gene expression, which needs further research in the furture.

In conclusion, this study demonstrated that GATA-1 bound to a specific domain of the human IRF-3 promoter *in vitro* and i*n vivo*. Additionally, down-regulated expression of GATA-1 protein might be responsible for the up-regulation of IRF-3 expression in lung adenocarcinoma cell lines which is in accordance with the observation in our Oncomine analysis and lung adenocarcinoma samples. Understanding this molecular mechanism may help to prove that IRF-3 is a useful target in the alternation of tumorigenesis in lung adenocarcinoma.

## Methods

### Cell Culture

A549, HeLa and HEK293 (ATCC) cells were maintained in Dulbecco’s modified Eagle’s medium (DMEM) with 10% fetal bovine serum (FBS) supplemented with penicillin and streptomycin. Human lung adenocarcinoma cell line H1299 were maintained in RPMI 1640 with 10% FBS, penicillin and streptomycin. Cells were cultured in a cell incubator and maintained at 5% CO_2_ and 37 °C.

### Analysis of Oncomine Data

To determine the expression pattern of IRF-3 and GATA-1 in lung adenocarcinoma, we used the datasets in the Oncomine database (https://www.oncomine.org). Oncomine is an online database consisting of previously published and publically available microarray data. The analysis enables multiple comparisons of gene expression (DNA or RNA) between different studies; the significance of the gene expression across the available studies was also taken into account^26,27^. IRF-3 and GATA1 gene were queried in the database and the results were filtered by selecting lung adenocarcinoma and Cancer vs. Normal Analysis. Comparison statistical analysis was conducted using Oncomine algorithms. Details of standardized normalization techniques and statistical calculations are provided on the Oncomine platform.

### Human Lung Adenocarcinoma Samples

Lung adenocarcinoma tissue and adjacent normal tissue were procured after surgical resection from patients with lung adenocarcinoma and stored at −70 °C in the biobank of the Translational Thoracic Oncology Laboratory at the Medical University of Vienna. All patients had given informed consent and the sample collection was approved by the Ethics Committees of the Medical University of Vienna (#904/2009). The investigation was conducted in accordance with human and ethical research principles of the Medical University of Vienna, Austria.

### Plasmids and siRNA

As we described before, transcriptional start site (TSS) of human IRF-3 promoter was set as +1 and the IRF-3 genomic DNA fragment −982 to +18, −149 to +18, −67 to +18 was inserted into the pGL3-Basic vector (Promega), respectively named as pGL3-982, pGL3-149, and pGL3-67^15^. The software TFSEARCH ver.1.3 (http://www.cbrc.jp/research/db/TFSEARCH.html) and Matlnspector (http://www.genomatix.de//matinspector.html) were used to search of consensus potential transcription factors target sites in IRF-3 promoter sequence. The GATA-1 binding site deletion promoter (pGL3-Del) was created by PCR from the cloned pGL3-149 plasmid, using site-directed mutagenesis kit (Takara). The forward primer was 5′-GGCCCAGCGTAGAAAGGGCGGAACGCT-3′; the reward primer was 5′-ACCCGGCCCAGTGCGCAGGCGCG-3′. For over-expression experiments, the plasmid pcDNA-GATA-1 was a kind gift from Dr. Feng Li (Li *et al*., 2015) and the corresponding control plasmid pcNDA3.1/Myc-HisA was purchased from Invitrogen (Carlsbad, CA, USA). The double-stranded siRNAs against human GATA-1 and control siRNA were synthesized and high-performance purified (GenePharma). The siRNA sequences used are the following (sense): 5′-AGUUGAGGCAGGGUAGAGCtt-3′ for GATA-1 siRNA; 5′-UUCUCCGAACGUGUCACGUtt-3′ for control siRNA.

### Transient Transfections and Luciferase Assays

A549 and HEK293 cells were seeded into 96-well plates (1.5 × 10^4^ cells/well). After 24 h, 100 ng luciferase reporter plasmids and 4 ng pRL-TK plasmid were transfected into cells according to the manufacturer’s instructions using Lipofectamine^TM^ 3000 (Invitrogen). For siRNA and overexpression assay, pGL3-149 or pGL3-Del plasmid was co-transfected with GATA-1 siRNA or pcDNA-GATA-1 into cells. Luciferase assay was performed 24 h post-transfection by a Dual-luciferase Reporter Assay System (Promega). Luciferase activity was normalized to the activity of pRL-TK. Results were representative of at least three independent experiments performed in triplicate.

### Reverse transcription and quantitative real-time PCR

Total RNA of cells and human lung tissue was reverse transcribed to cDNA with equal RNA volume (1 μg) using the PrimeScript RT Master Mix Perfect Real Time kit (Takara). Real-time PCR was performed using Applied Biosystems Step One Plus Real-Time PCR System, using cDNA and SYBR Green (TaKaRa) detection. Primer sequences for detection of IRF-3 mRNA expression were synthesized as 5′-GGACCCTCACGACCCACATA-3′ (sense) and 5′-CCATGTTACCCAGTAACTCATCCAG-3′ (anti-sense). All experiments were repeated at least three times. Gene expression levels were calculated relative to the housekeeping gene GAPDH.

### Western Blot

Total protein was extracted from frozen tissues or cells using a Total Protein Extraction Kit (Keygentec, China). Protein concentrations were determined by the BCA Protein Assay Kit (Beyotime). Equal amount of denatured protein were separated by 10% SDS-PAGE and transferred to a PVDF membrane (Millipore). The membrane were blocked with 5% nonfat milk in TBST (containing 0.1% Tween-20) and then incubated with anti-IRF-3 (Abcam), anti-GATA-1 (Abcam), or anti-GAPDH antibody (Santa Cruz) at 4 °C overnight at a dilution of 1:2000–1:4000, followed by anti-rabbit IgG (Cell signaling) or anti-mouse IgG (Jackson ImmunoResearch Laboratories) at a dilution of 1:5000. The intensity of bands was measured by chemiluminescence (ECL) system.

### EMSA

Nuclear extracts of A549 cells were prepared using a NE-PER (Thermo Scientific) according to the manufacturer’s protocol. The double-stranded oligonucleotides were synthesized (Invitrogen) as following: wild-type 5′-biotinylated double-stranded oligonucleotides containing the GATA-1 binding motif are: 5′-GGCCGGGTCGATAACCGGCCCA-3′ and the complement. Detection of the GATA-1 oligonucleotide complex was performed using the Light Shift Chemiluminescent EMSA Kit (Thermo Scientific). Nuclear protein extract was incubated in a buffer containing 10 mM Tris, 50 mM KCl, 1 mM DTT (pH 7.5), 7.5% glycerol, and 75 ng/ml poly(dI-dC) for 20 min at room temperature. Then, 1 μl of the biotinylated oligonucleotides were added to the reaction mixtures and incubated at room temperature for 40 min. For competition experiments, unlabeled probes were added to the reaction mixture 20 min before addition of the labeled probe. Finally, the DNA-protein complexes were resolved on a 6% polyacrylamide gel in 0.5% TBE buffer for 1 h.

### ChIP

ChIP assays were performed with the EZ-Magna ChipTM A kit (Millipore) following the manufacturer’s instructions. A total of 1.5 × 10^7^ A549 cells were cross-linked with 1% formaldehyde at room temperature for 10 min. Sonication was performed on ice to get 200 to1000 bp DNA fragments. The chromatin was then immunoprecipitated with anti-IgG antibody (Millipore) and anti-GATA-1 antibody. After reverse cross-linking and DNA purification, DNA from input (1:10 diluted) or immunoprecipitated samples were assayed by semi-quantitative PCR, and the products were separated by 2% agarose gel electrophoresis. Forward and reverse primers for the GATA-1 binding site were 5′-GTACTGAACTCGTAC-3′ and 5′-GCCCTTTTTTGGGTTTCC-3′.

## Electronic supplementary material

Supplementary information
