# Author Correction: Down-regulated GATA-1 up-regulates interferon regulatory factor 3 in lung adenocarcinoma

**DOI:** 10.1038/s41598-018-22517-0

**Published:** 2018-03-06

**Authors:** Lu-Lu Wang, Zheng-Sen Chen, Wen-Di Zhou, Jin Shu, Xiao-Hua Wang, Rui Jin, Li-Li Zhuang, Mir Alireza Hoda, Hao Zhang, Guo-Ping Zhou

**Affiliations:** 1 Dpartment of Pediatrics, The First Affiliated Hospital, Nanjing Medical University, Nanjing, Jiangsu Province China; 2 Department of Urology, The Second Affiliated Hospital, Nanjing Medical University, Nanjing, Jiangsu Province China; 3 Dpartment of Pediatrics, Huai’an First People’s Hospital, Nanjing Medical University, Huai’an, Jiangsu Province China; 4 Department of Pediatric Respiration, Affiliated Wuxi People’s Hospital, Nanjing Medical University, Wuxi, Jiangsu Province China; 5 Dpartment of Pediatrics, Nanjing First Hospital, Nanjing Medical University, Nanjing, Jiangsu Province China; 6 Translational Thoracic Oncology Laboratory, Division of Thoracic Surgery, Department of Surgery, Comprehensive Cancer Center, Medical University Vienna, Vienna, Austria; 7 Department of Thoracic and Cardiovascular Surgery, Affiliated Hospital of Xuzhou Medical University, Xuzhou, Jiangsu Province China

Correction to: *Scientific Reports* 10.1038/s41598-017-02700-5, published online 31 May 2017

The original version of this Article contained an error in the order of corresponding authors. This error has now been corrected in the PDF and HTML versions of the Article.

