# Supplementary material for: Down-regulated GATA-1 up-regulates interferon regulatory factor 3 in lung adenocarcinoma
Source: Sci Rep. 2017 May 31;7:2551. doi: 10.1038/s41598-017-02700-5 (PMC5451405; doi:10.1038/s41598-017-02700-5)

SUPPLEMENTARY INFORMATION

Down-regulated GATA-1 up-regulates interferon regulatory factor 3 in lung adenocarcinoma

Lu-Lu Wang[1], Zheng-Sen Chen[2], Wen-Di Zhou[3], Jin Shu[4], Xiao-Hua Wang[5], Rui Jin[1], Li-Li Zhuang[1], Mir Alireza Hoda[6], Hao Zhang[7]*, Guo-Ping Zhou[1]*

[1] Dpartment of Pediatrics, The First Affiliated Hospital, Nanjing Medical University, Nanjing, Jiangsu Province, China,
[2] Department of Urology, The Second Affiliated Hospital, Nanjing Medical University, Nanjing, Jiangsu Province, China,
[3] Dpartment of Pediatrics, Huai'an First People's Hospital, Nanjing Medical University, Huai'an, Jiangsu Province, China,
[4] Department of Pediatric Respiration, Affiliated Wuxi People's Hospital, Nanjing Medical University, Wuxi, Jiangsu Province, China,
[5] Dpartment of Pediatrics, Nanjing First Hospital, Nanjing Medical University, Nanjing, Jiangsu Province, China,
[6] Translational Thoracic Oncology Laboratory, Division of Thoracic Surgery, Department of Surgery, Comprehensive Cancer Center, Medical University Vienna, Vienna, Austria,
[7] Department of Thoracic and Cardiovascular Surgery, Affiliated Hospital of Xuzhou Medical University, Xuzhou, Jiangsu Province, China.

Corresponding to: Guo-Ping Zhou, Email: guopingzhou@aliyun.com
Hao Zhang, Email: zhanghao@xzhmu.edu.cn

Supplementary Figure S1. Gel from the article.

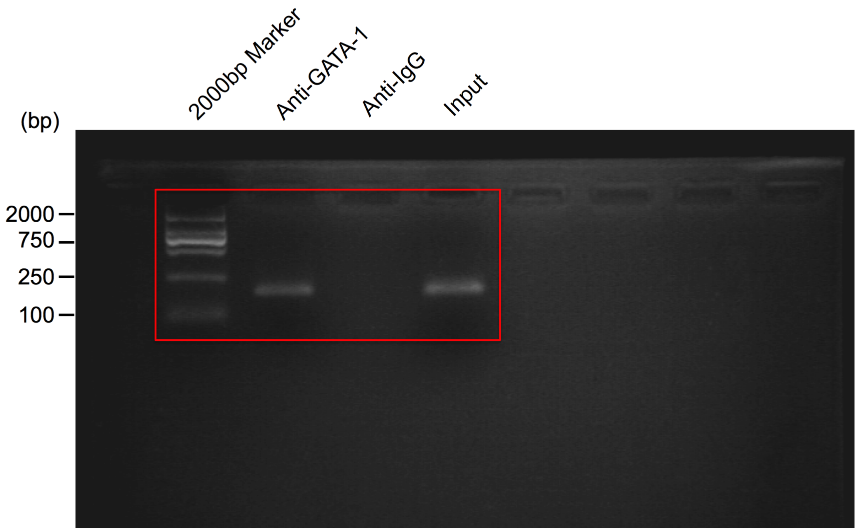

Supplementary Figure S2. Western Blot from the article.

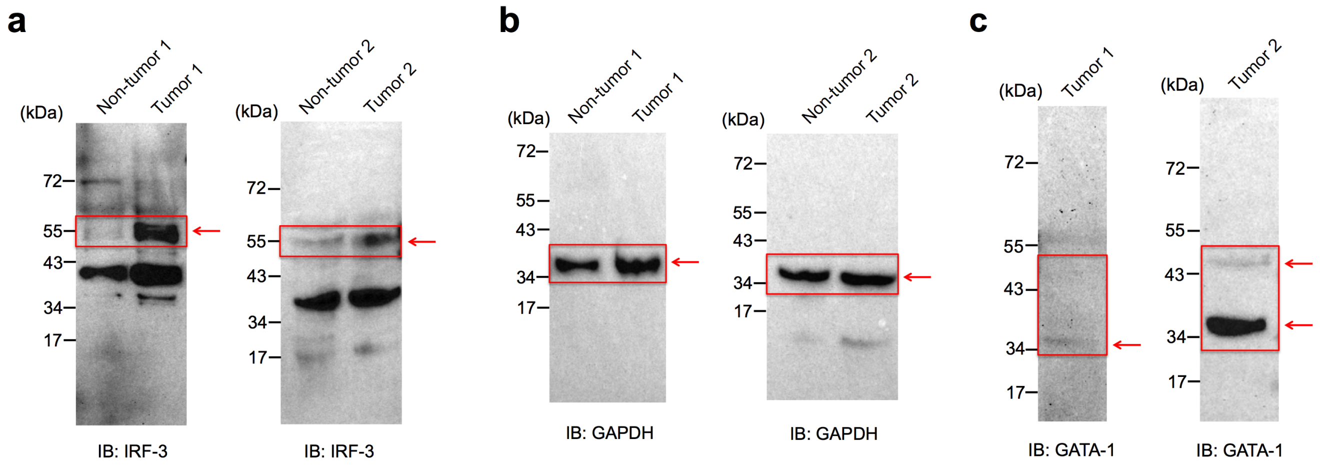

Supplementary Figure S3. Western Blot from the article.

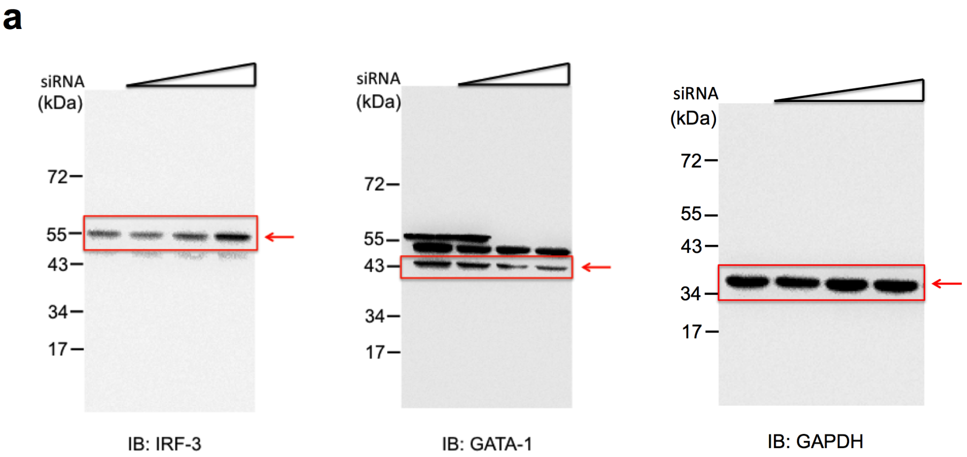

Supplementary Figure S4.

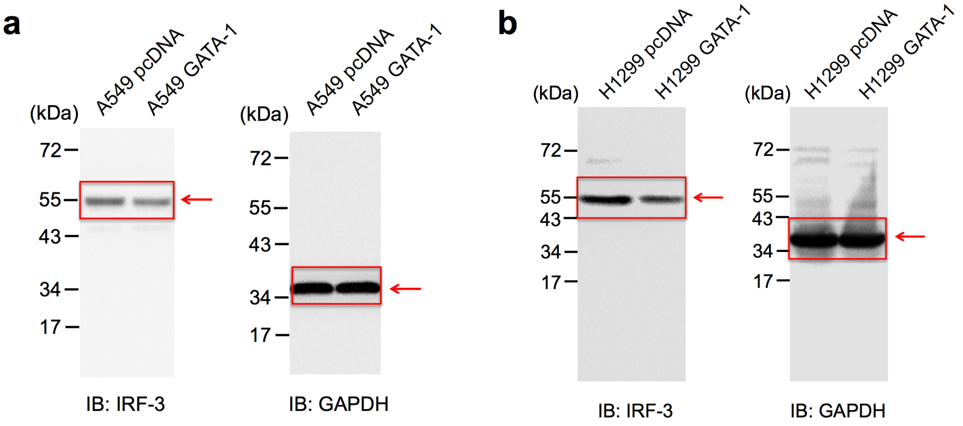

Supplementary Figure S5. Overexpression of GATA-1 level decreases IRF-3 protein expression. (a) IRF-3 protein expression was decreased by overexpressed GATA-1 in HEK293 and HeLa cells. GAPDH was used as a loading control. Full-length blots are also presented.

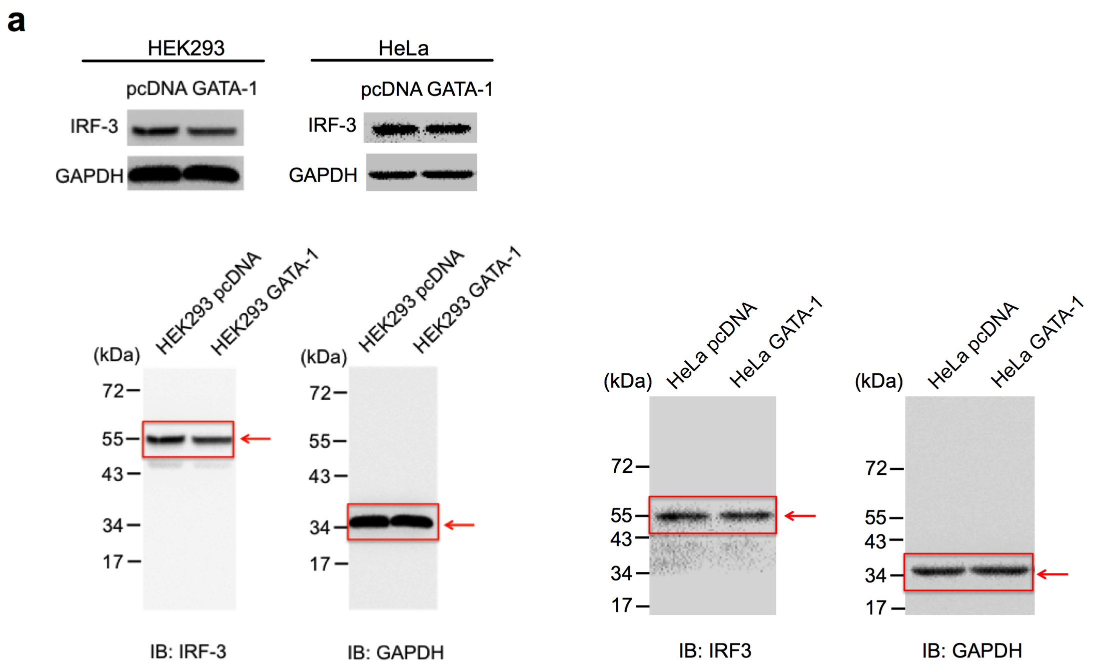

Supplement: Supplementary file 1 — Supplementary information [file 41598_2017_2700_MOESM1_ESM.pdf]
